# Supplementary material for: Velocity of Sargassum migration in the Caribbean observed with Landsat 8/9 and Sentinel 2 A/B imagery
Source: PLoS One. 2025 Mar 12;20(3):e0319391. doi: 10.1371/journal.pone.0319391 (PMC11902290; doi:10.1371/journal.pone.0319391)

PLOS ONE

Velocity of Sargassum migration in the Caribbean observed with Landsat 8/9 and Sentinel 2 A/B imagery.

--Manuscript Draft--

**Manuscript Number:**	PONE-D-24-29369
**Article Type:**	Research Article
**Full Title:**	Velocity of Sargassum migration in the Caribbean observed with Landsat 8/9 and Sentinel 2 A/B imagery.
**Short Title:**	Velocity of Sargassum migration in the Caribbean observed with satellite imagery.
**Corresponding Author:**	Jorge Iván Euan-Avila, PhD<br>CINVESTAV: Centro de Investigacion y de Estudios Avanzados del Instituto Politecnico Nacional<br>Merida, Yucatan MEXICO
**Keywords:**	velocity; Sargassum; Satellite; migration; Caribbean
**Abstract:**	Summary<br><br>Landsat 8/9 (L89) and Sentinel-2 A/B (S2) imagery were used to track velocity migration of Sargassum aggregations. Displacement characteristics provides valuable information for making preventive decisions and planning harvest the biomass. Images L89 and S2 are sometimes acquired the same day and a few minutes apart. Sargassum landmark identification was performed manually on enhanced RGB composite images using quotient indices. A review of images between 2019 and 2023 was performed to select rafts that were distinguishable in both times. Geographic positions were recorded to determine distance traveled, direction, and speed. Pairs of 279 patches were found on 21 coincident dates. Ninety eight percent of Sargassum rafts traveled between 200 m and 1700 m in a time frame of 14 to 26 minutes with an average speed of 0.63 m/s, a standard deviation of 0.24 m/s, a minimum of 0.15 m/s, and a maximum of 1.40m/s. Dominant directions were 34% NW, 23% WNW, 14% NNW, 14% W and 6% N. HYCOM ocean currents showed a positive correlation with Sargassum drift, and translation rates are also consistent with surface drifter data. The use of L89 and S2 satellite imagery as an early warning system, in conjunction with current and wind data, may help anticipate the arrival of Sargassum in coastal areas.
**Order of Authors:**	Jorge Iván Euan-Avila, PhD
	Héctor Hernández Nuñez
**Additional Information:**	
**Question**	**Response**
**Financial Disclosure**<br><br>Enter a financial disclosure statement that describes the sources of funding for the work included in this submission. Review the submission guidelines for detailed requirements. View published research articles from *PLOS ONE* for specific examples.<br><br>This statement is required for submission and **will appear in the published article** if the submission is accepted. Please make sure it is accurate.	The author(s) received no specific funding for this work.

Powered by Editorial Manager® and ProduXion Manager® from Aries Systems Corporation

**Funded studies**<br>Enter a statement with the following details:<br>• Initials of the authors who received each award<br>• Grant numbers awarded to each author<br>• The full name of each funder<br>• URL of each funder website<br>• Did the sponsors or funders play any role in the study design, data collection and analysis, decision to publish, or preparation of the manuscript?<br><br>Did you receive funding for this work?	
**Competing Interests**<br><br>Use the instructions below to enter a competing interest statement for this submission. On behalf of all authors, disclose any competing interests that could be perceived to bias this work—acknowledging all financial support and any other relevant financial or non-financial competing interests.<br><br>This statement is required for submission and **will appear in the published article** if the submission is accepted. Please make sure it is accurate and that any funding sources listed in your Funding Information later in the submission form are also declared in your Financial Disclosure statement.<br><br>View published research articles from *PLOS ONE* for specific examples.	The authors have declared that no competing interests exist

Powered by Editorial Manager® and ProduXion Manager® from Aries Systems Corporation

**NO authors have competing interests**<br><br>Enter: *The authors have declared that no competing interests exist.*<br><br>**Authors with competing interests**<br><br>Enter competing interest details beginning with this statement:<br><br>*I have read the journal's policy and the authors of this manuscript have the following competing interests: [insert competing interests here]*<br><br>* typeset	
**Ethics Statement**<br><br>Enter an ethics statement for this submission. This statement is required if the study involved:<br><br>• Human participants<br>• Human specimens or tissue<br>• Vertebrate animals or cephalopods<br>• Vertebrate embryos or tissues<br>• Field research<br><br>Write "N/A" if the submission does not require an ethics statement.<br><br>General guidance is provided below. Consult the submission guidelines for detailed instructions. **Make sure that all information entered here is included in the Methods section of the manuscript.**	N/A

**Format for specific study types**<br><br>**Human Subject Research (involving human participants and/or tissue)**<br>• Give the name of the institutional review board or ethics committee that approved the study<br>• Include the approval number and/or a statement indicating approval of this research<br>• Indicate the form of consent obtained (written/oral) or the reason that consent was not obtained (e.g. the data were analyzed anonymously)<br><br>**Animal Research (involving vertebrate animals, embryos or tissues)**<br>• Provide the name of the Institutional Animal Care and Use Committee (IACUC) or other relevant ethics board that reviewed the study protocol, and indicate whether they approved this research or granted a formal waiver of ethical approval<br>• Include an approval number if one was obtained<br>• If the study involved *non-human primates*, add *additional details* about animal welfare and steps taken to ameliorate suffering<br>• If anesthesia, euthanasia, or any kind of animal sacrifice is part of the study, include briefly which substances and/or methods were applied<br><br>**Field Research**<br><br>Include the following details if this study involves the collection of plant, animal, or other materials from a natural setting:<br>• Field permit number<br>• Name of the institution or relevant body that granted permission	
**Data Availability**<br><br>Authors are required to make all data underlying the findings described fully available, without restriction, and from the time of publication. PLOS allows rare exceptions to address legal and ethical concerns. See the PLOS Data Policy and FAQ for detailed information.	Yes - all data are fully available without restriction

<table>
<tr>
<td>
A Data Availability Statement describing where the data can be found is required at submission. Your answers to this question constitute the Data Availability Statement and will be published in the article, if accepted.
Important: Stating ‘data available on request from the author’ is not sufficient. If your data are only available upon request, select ‘No’ for the first question and explain your exceptional situation in the text box.
Do the authors confirm that all data underlying the findings described in their manuscript are fully available without restriction?
</td>
<td></td>
</tr>
<tr>
<td>
Describe where the data may be found in full sentences. If you are copying our sample text, replace any instances of XXX with the appropriate details.

If the data are held or will be held in a public repository, include URLs, accession numbers or DOIs. If this information will only be available after acceptance, indicate this by ticking the box below. For example: All XXX files are available from the XXX database (accession number(s) XXX, XXX.).
If the data are all contained within the manuscript and/or Supporting Information files, enter the following: All relevant data are within the manuscript and its Supporting Information files.
If neither of these applies but you are able to provide details of access elsewhere, with or without limitations, please do so. For example:

Data cannot be shared publicly because of [XXX]. Data are available from the XXX Institutional Data Access / Ethics Committee (contact via XXX) for researchers who meet the criteria for access to confidential data.

The data underlying the results presented in the study are available from (include the name of the third party

</td>
<td>All relevant data are within the manuscript and its Supporting Information files.</td>
</tr>
</table>

and contact information or URL).<br>• This text is appropriate if the data are owned by a third party and authors do not have permission to share the data.<br><br>* typeset	
Additional data availability information:	

Manuscript Click here to access/download;Manuscript;Manuscript.docx

Velocity of *Sargassum* migration in the Caribbean observed with Landsat 8/9 and Sentinel 2 A/B imagery.

Héctor Hernández Nuñez, Jorge Iván Euán-Avila

Center for Research and Advanced Studies of the National Polytechnic Institute (CINVESTAV).

Key words: velocity, *Sargassum*, satellite, migration, Caribbean

Summary

Landsat 8/9 (L89) and Sentinel-2 A/B (S2) imagery were used to track velocity migration of *Sargassum* aggregations. Displacement characteristics provides valuable information for making preventive decisions and planning harvest the biomass. Images L89 and S2 are sometimes acquired the same day and a few minutes apart. *Sargassum* landmark identification was performed manually on enhanced RGB composite images using quotient indices. A review of images between 2019 and 2023 was performed to select rafts that were distinguishable in both times. Geographic positions were recorded to determine distance traveled, direction, and speed. Pairs of 279 patches were found on 21 coincident dates. Ninety eight percent of Sargassum rafts traveled between 200 m and 1700 m in a time frame of 14 to 26 minutes with an average speed of 0.63 m/s, a standard deviation of 0.24 m/s, a minimum of 0.15 m/s, and a maximum of 1.40m/s. Dominant directions were 34% NW, 23% WNW, 14% NNW, 14% W and 6% N. HYCOM ocean currents showed a positive correlation with *Sargassum* drift, and translation rates are also consistent with surface drifter data. The use of L89 and S2 satellite imagery as an early warning system, in conjunction with current and wind data, may help anticipate the arrival of *Sargassum* in coastal areas.

Introduction

The massive arrival of pelagic *Sargassum* in the Caribbean has become an environmental concern due to its potential impact on mangroves, seagrasses and coral reefs (Johns et al., 2020; Chávez et al., 2020; van Tussenbroek et al., 2017; Robledo et al., 2021). A calamity for the tourism and fishing sectors by modifying landscapes and ecosystems through the alteration of the visual quality of sandy beaches, reduction of water quality, and negative changes in the habitats of species for recreational and fishing activities (Rodríguez-Martínez et al., 2023; Chávez et al., 2020). At the same time, it has been seen as an opportunity to be used for multiple purposes, including animal food, pharmaceutical products, and energy generation (Milledge and Harvey, 2016; Vázquez-Delfín et al., 2019; López Miranda et al., 2021). The above has motivated studies for its detection and quantification (Cuevas et al., 2018; Gower et al., 2006), as well as to determine the characteristics of its migration in open waters, an aspect of particular interest for decision making for preventive measurements and harvesting operations.

Pelagic *Sargassum* is a marine algae that drifts in the ocean and has highly branched thalli, which can be up to 100 cm in length, with leaves and vesicles that allow the thalli to float either individually and dispersed, or in aggregations of highly variable shape and size (Ody et al., 2019; Uribe-Martínez et al., 2022; Vázquez-Delfín et al., 2020). Due to their buoyancy characteristics, their distribution is largely at the mercy of currents and winds, forming a variety of aggregations that can extend for tens of kilometers, presenting an opportunity for remote sensing technologies to detect them. In their detection, satellite remote sensing through different platforms has contributed monitoring using low spatial resolution but highly synoptic platforms such as MODIS, VIIRS, and Sentinel 3 with pixel sizes of hundreds of meters and temporal resolutions of hours, and high resolution platforms such as L89 and S2 with pixels up to tens of meters and temporal resolutions of days (Ody et al., 2019; Cuevas et al., 2018; Hu et al., 2015; Wang et al., 2019).

Due to their orbital design, the Landsat and Sentinel platforms capture scenes very close in time at certain times. Taking advantage of this capability, the objective of this work is to determine the displacement of *Sargassum* rafts using satellite imagery acquired at short time intervals to determine their displacement, and estimate their speed and direction in a manner analogous to drift buoys.

Study Area

It is located in the Caribbean, specifically in the Western Caribbean subregion. It is bounded by L89 tracks 17 and 18, scenes 47 and 48 according to the world reference system (WRS) of the Landsat program (https://landsat.gsfc.nasa.gov/about/the-worldwide-reference-system/) and those tiles corresponding to S2 (https://hls.gsfc.nasa.gov/products-description/tiling-system/) (Fig. 1). The area is strongly influenced by the Yucatan Current and the gyres which occur south of Cuba, as well as by the currents of the Caymanes Basin and those of the Gulf of Honduras (Centurioni & Niiler, 2003b) (Fig. 2). The winds have a regional pattern in which the NE or trade winds dominate, which are derived from the Earth's general atmospheric circulation system due to the Atlantic anticyclone (Cuevas Jiménez et al., 2014). The platform in its submerged topography at 19°N latitude is relatively short, extending about 60km and reaching a depth of 1000 m, at the limits of the study area at a distance of 300km at a depth of 4250 m (Gebco 2021). The main elevations on the shelf are the island of Cozumel, the Chinchorro Bank and the atolls off the coast of Belize. This coastline from Belize to Cancun in Mexico is an important part of the Mesoamerican reef and *Sargassum* transit to the Gulf of Mexico where, according to estimates made by the Oceanographic Institute of the Gulf and Caribbean Sea in 2022 a 63,000 ton of *Sargassum* have been detected floating in the area (SEMAR, 2024).

Figure 1. The study area in the Caribbean Sea and the number of *Sargassum* raft samples taken from the L89 and S2 images.

Figure 2. Ocean current pattern model HYCOM (July 20, 2023). https://www.hycom.org/

Method

Floating *Sargassum* rafts have been detected with L89 and L2 images acquired the same day a few minutes apart from one platform to the other; in general, the L89 satellites are ahead in time and the S2 satellites follow a few minutes later. Images where satellites encounters occur were identified between the years 2019 to 2023. In order to identify the rafts, RGB composite images based on quotient indices were created for both platforms, and a semi-automatic segmentation process was used by the analyst to select and match the rafts.

The L89 scenes are acquired during a north-south sweep at a local time around 10:25 am (16:25 GMT) (https://www.usgs.gov/faqs/how- can-i-find-acquisition-time-landsat-scene) and around 10:30 am for S2 (https://sentinels.copernicus.eu/web/sentinel/missions/sentinel-2/satellite-description/orbit). The date and time of capture are relative to the center of the scene according to the metadata. Time and *Sargassum* traveled distance is used to estimate speed.

The selection of the *Sargassum* rafts consisted of identifying those that had particular characteristics in their shape and in conjunction with other elements in the vicinity, such as other *Sargassum* formations, clouds, and the shadow of the clouds, with the purpose of increasing the certainty that the raft or part of it would be observable in both images.

Once the object was identified, the correspondence was established by selecting a pixel either from the central part of the raft, a corner or protrusion of the contour in each image and recording the coordinates (Lon, Lat) in the UTM-WGS84 system at both times (Figure 3).

Figure 3. Point (Lat, Lon) selection on *Sargassum* rafts in Landsat (P17_1 - time 1) and Sentinel 2 (P17_2 - time 2).

The ocean current data (u, v) were downloaded from the Global Ocean Forecasting System (GOFS) 3.1 platform and produced with the HYCOM (HYbrid Coordinate Ocean Model) model (https://www.hycom.org/). The resolution of this model (GOFS 3.1, Global Analysis) uses a grid of 0.08° longitude x 0.04° latitude and covers 80S to 90N. The speed and direction values of *Sargassum* in the images were compared with those of the models generated at the time closest to 16:00:00 UTC, the time of the satellite pass. Directions in degrees were converted in 16 standard rose plot with North at 0°. Class frequencies were compared to corroborate how well *Sargassum* and current directions match. 5. Statistical data were processed with InfoStat/L (Di Rienzo, at al., 2020).

Results

Sargassum

A total of 279 pairs of rafts were identified in 22 L89 and S2 images acquired in the same date in the years 2019, 2021, 2022 and 2023 (see Table 1). Total samples of *Sargassum* drift vectors are shown in figure (4).

Table 1. Months per year where pairs of rafts were founded in Landsat 8/9 and Sentinel 2 (A/B) satellites images between the years 2019-2023.

Year	Month	Samples
2023	January, February, March, April, May, June, July	150
2022	August, September	39
2021	October	40
2020	n/a	0
2019	January, February, April, May	50
	Total	279

Figure 4. Observed total samples of *Sargassum* drift speed and direction.

Ninety-eight percent of the Sargassum rafts traveled between 200 m and 1700 m in a range time of 14 to 26 minutes. Five rafts traveled between 22 and 40 km in 24 hours. Velocities ranged from 0.15 m/s to 1.4 m/s (see Table 2). According to the twelve S2 tiles into which the area was divided, Table 3 and Figure 5 show Sargassum velocity statistics and Figure 6 illustrates velocity similarities by tile, the QGE (1.39 m/s) and QFG (0.88 m/s) resulted with the highest velocities, while the QEE (0.44 m/s) and QED (0.38 m/s) resulted with the lowest values.

Table 2. Total samples *Sargassum* speed statistics (m/s).

n	Mean	std	Min	Max	Median
279	0.64	0.24	.15	1.40	0.64

Table 3. *Sargassum* speeds statistics by tiles (m/s).

Tile	n	Mean	Std	Min	Max	Median
QDE	16	0.63	0.17	0.27	0.89	0.68
QDF	21	0.58	0.18	0.28	0.94	0.56
QDG	4	0.64	0.05	0.57	0.68	0.66
QED	12	0.38	0.12	0.18	0.57	0.38
QEE	27	0.43	0.18	0.15	0.85	0.44
QEF	35	0.64	0.19	0.27	1.03	0.65
QEG	25	0.55	0.2	0.2	1.05	0.57
QFE	27	0.54	0.16	0.22	0.77	0.58
QFF	71	0.71	0.19	0.26	1.12	0.69
QFG	10	0.88	0.25	0.29	1.2	0.88
QGE	7	1.36	0.06	1.24	1.4	1.39
QGF	24	0.71	0.11	0.55	0.85	0.71

Figure 5. Standard Box-Plot of the *Sargassum* speed by tiles.

Figure 6. *Sargassum* speed similarities among tiles (Cophenetic correlation = 0.953)

Direction in which the *Sargassum* moves dominate with 34% towards the NW, 23% WNW, 14% in the NNW direction, 14% W and 6% N (Figure 7). Table 4 shows the statistics of the migration directions within each of the tiles.

Table 4. Sargassum directions statistics by tile (0° N)

Tile	n	Mean	Std	Min	Max	Median
QDE	16	313.25	17.46	267	325	319
QDF	21	297.86	77.44	1	357	309
QDG	4	91.25	179.17	1	360	2
QED	12	250.17	38.52	181	283	271
QEE	27	277.11	34.75	179	330	281
QEF	35	307.54	35.71	150	350	310
QEG	25	304.56	86.61	16	356	328
QFE	27	286.96	24.89	250	318	294
QFF	71	304.04	20.05	264	341	305
QFG	10	289.2	12.84	273	313	286.5
QGE	7	291.86	3.63	286	296	291
QGF	24	328.92	16.61	309	358	325.5

Figure 7. Wind-rose of velocity for total floating *Sargassum* samples.

Ocean currents

Statistics of speed of ocean currents were obtained from the closest pixel of HYCOM models for the same day satellite images at 16:00 UTC where Sargassum was sampled (Table 5 and Table 6). The mean difference indicates that the speed of Sargassum is 0.13 m/s higher than the ocean currents and the maximum speed value was also observed in Sargassum with 1.4 m/s. The statistics of direction by tile is shown in Table 7. Ocean currents directions show a pattern with dominant directions in NNW (27%), NW (24%) and WNW (13%) (Figure 8).

Table 5. Total samples HYCOM Current speed statistics (m/s).

n	Mean	std	Min	Max	Median
279	0.51	0.20	0.01	1.01	0.53

Table 6. Current HYCOM speed statistics by tile (m/s)

Tile	n	Mean	Std	Min	Max	Median
QDE	16	0.48	0.05	0.4	0.56	0.5
QDF	21	0.58	0.19	0.2	0.82	0.6
QDG	4	0.51	0.19	0.32	0.74	0.48
QED	12	0.26	0.03	0.21	0.3	0.26
QEE	27	0.27	0.13	0.06	0.54	0.27
QEF	35	0.46	0.25	0.04	0.82	0.55
QEG	25	0.55	0.16	0.22	0.89	0.55
QFE	27	0.59	0.08	0.42	0.73	0.59
QFF	71	0.55	0.2	0.01	1.01	0.56
QFG	10	0.48	0.22	0.18	0.74	0.5
QGE	7	0.83	0.09	0.68	0.93	0.85
QGF	24	0.59	0.06	0.51	0.66	0.61

Figure 8. Wind-rose of velocity for the ocean HYCOM currents.

Table 7. Currents HYCOM direction statistics by tiles (0° N).

Tile	n	Mean	Std	Min	Max	Median
QDE	16	316.69	23.22	259.32	341.38	320.16
QDF	21	222.13	137.46	7.82	342.07	306.68
QDG	4	12.16	7.82	5.69	23.24	9.85
QED	12	277.89	43.3	182.83	309.98	289.13
QEE	27	290.09	35.57	181.24	341.86	291.42
QEF	35	266.53	112.2	6.43	355.3	304.38
QEG	25	208.21	166.88	0.17	359.1	332.97
QFE	27	309.09	35.57	247.92	355.42	325.04
QFF	71	305.53	39.49	124.04	349.18	321.71
QFG	10	291.78	38.1	233.59	326.45	312.13
QGE	7	274.54	4.41	267.21	279.19	274.84
QGF	24	330.85	7.3	319.38	339.4	328.76

Discussion and conclusions

The speeds found are similar to those observed by surface drifters reported by Centurioni, et al., (2003) and Richardson (2004) with strongest instantaneous speeds above 0.8 m/s located in the Yucatan current with values of up to 1.0 m/s at 20o N latitude. The Sargassum speed and the ocean speed modeled by HYCOM is positive correlated (R2 = 0.15, p-value = .0001), as well as the generalized transport of pelagic Sargassum towards the NW and its neighbors NNW and WNW are consistent to HYCOM prevalent marine currents in this area. This transport pattern causes tons of Sargassum biomass to reach on the coastal areas of the Mexican state of Quintana Roo, generating large accumulations in artificial net barriers placed offshore at some distance from the coastline and on the unprotected beaches. Monitoring paths 17 and 18 of L89 and corresponding S2 images allowed to detect the presence of Sargassum and to estimate a general average speed of 0.63 m/s (2.2 km/h). Together with the dominant observed directions in this work, scenarios of possible arrival times of Sargassum rafts at different distances and locations can be constructed. The joint sampling frequency between L89 and S2 with a temporal resolution of three to four days at a distance between 150 and 180 km from the coast will provide an early warning for the presence of Sargassum. In addition, and with the support of weather products such as winds and ocean currents provided by various environmental platforms, can reduce the uncertainty of arrivals and help support coastal management needs. The infrequent opportunities that platforms L89 and S2 can offer together to obtain close temporal (minutes) shots, the high presence of clouds in the area, the rapid deformation that rafts undergo over time, and the natural variability of Sargassum abundance and distribution throughout the year are the main constraints to integrate a robust data base. In this sense, the spatio-temporal data collected do not have a probabilistic sampling base, but the uncertainty in our measurements can be reduced by adding new observations in future years.

Author Contributions Héctor Hernández Nuñez: Conceptualization, Investigation, Methodology, Data curation, Data analysis, Writing – original draft, Visualization. Jorge Iván Euán Avila: Conceptualization, Supervision, Data analysis, Writing – review & editing.

Funding This work was supported by Center for Research and Advanced Studies of the National Polytechnic Institute (CINVESTAV) and Consejo Nacional de Humanidades Ciencia y Tecnología (CONAHCYT – SNII)

Data Availability The datasets generated during the current study are available from the corresponding author on reasonable request.

Declarations

Competing Interests, the authors declare no competing interests.

Ethical Approval This declaration is “not applicable

References

1. Centurioni, L. R., & Niiler, P. P. (2003). On the surface currents of the Caribbean Sea. Geophysical Research Letters, 30(6). https://doi.org/10.1029/2002GL016231
2. Chávez, V., Uribe-Martínez, A., Cuevas, E., Rodríguez-Martínez, R. E., van Tussenbroek, B. I., Francisco, V., Estévez, M., Celis, L. B., Monroy-Velázquez, L. V., Leal-Bautista, R., Álvarez-Filip, L., García-Sánchez, M., Masia, L., & Silva, R. (2020). Massive Influx of Pelagic Sargassum spp. on the Coasts of the Mexican Caribbean 2014–2020: Challenges and Opportunities. Water, 12(10), 2908. https://doi.org/10.3390/w12102908
3. Cuevas, E., Uribe-Martínez, A., & Liceaga-Correa, M. de los Á. (2018). A satellite remote-sensing multi-index approach to discriminate pelagic Sargassum in the waters of the Yucatan Peninsula, Mexico. International Journal of Remote Sensing, 39(11), 3608–3627. https://doi.org/10.1080/01431161.2018.1447162
4. Cuevas-Jimenez, A., Orellana-Lanza y Euan-Avila, J. I. (2014) Capítulo 7: Patrones locales del viento en La costa del estado de Yucatán, un espacio de reflexión sobre la relación sociedad-naturaleza, en el contexto de su ordenamiento ecológico territorial, J. I. Euán-Ávila, A. García de Fuentes, M. A. Liceaga-Correa y A. Munguía Gil (eds.). Plaza y Valdés, México. Vol. I, pag.137- 149.
5. Di Rienzo J.A., Casanoves F., Balzarini M.G., Gonzalez L., Tablada M., Robledo C.W. InfoStat versión 2020. Centro de Transferencia InfoStat, FCA, Universidad Nacional de Córdoba, Argentina. URL http://www.infostat.com.ar
6. García-Sánchez, M., Graham, C., Vera, E., Escalante-Mancera, E., Álvarez-Filip, L., & van Tussenbroek, B. I. (2020). Temporal changes in the composition and biomass of beached pelagic Sargassum species in the Mexican Caribbean. Aquatic Botany, 167, 103275. https://doi.org/10.1016/j.aquabot.2020.103275
7. Gärtner, Philipp (2018) “When Landsat-8 (almost) meets Sentinel-2”, https://philippgaertner.github.io/2018/07/landsat-meets-sentinels/
8. Gebco (2021). https://www.gebco.net/data_and_products/gridded_bathymetry_data/gebco_2021/
9. Gower, J., Hu, C., Borstad, G., & King, S. (2006). Ocean Color Satellites Show Extensive Lines of Floating Sargassum in the Gulf of Mexico. IEEE Transactions on Geoscience and Remote Sensing, 44(12), 3619–3625. https://doi.org/10.1109/TGRS.2006.882258
10. Gower, J., Young, E., & King, S. (2013). Satellite images suggest a new Sargassum source region in 2011. Remote Sensing Letters, 4(8), 764–773. https://doi.org/10.1080/2150704X.2013.796433
11. Hu, C., Feng, L., Hardy, R. F., & Hochberg, E. J. (2015). Spectral and spatial requirements of remote measurements of pelagic Sargassum macroalgae. Remote Sensing of Environment, 167, 229–246. https://doi.org/10.1016/j.rse.2015.05.022
12. Johns, E. M., Lumpkin, R., Putman, N. F., Smith, R. H., Muller-Karger, F. E., T. Rueda-Roa, D., Hu, C., Wang, M., Brooks, M. T., Gramer, L. J., & Werner, F. E. (2020). The establishment of a pelagic Sargassum population in the tropical Atlantic: Biological consequences of a basin-scale long distance dispersal event. Progress in Oceanography, 182, 102269. https://doi.org/10.1016/j.pocean.2020.102269

13. López Miranda, J. L., Celis, L. B., Estévez, M., Chávez, V., van Tussenbroek, B. I., Uribe-Martínez, A., Cuevas, E., Rosillo Pantoja, I., Masia, L., Cauich-Kantun, C., & Silva, R. (2021). Commercial Potential of Pelagic Sargassum spp. in Mexico. Frontiers in Marine Science, 8. https://doi.org/10.3389/fmars.2021.768470
14. Milledge, J., & Harvey, P. (2016). Golden Tides: Problem or Golden Opportunity? The Valorisation of Sargassum from Beach Inundations. Journal of Marine Science and Engineering, 4(3), 60. https://doi.org/10.3390/jmse4030060
15. Miron, P., Olascoaga, M. J., Beron-Vera, F. J., Putman, N. F., Triñanes, J., Lumpkin, R., & Goni, G. J. (2020). Clustering of Marine-Debris- and Sargassum-Like Drifters Explained by Inertial Particle Dynamics. Geophysical Research Letters, 47(19). https://doi.org/10.1029/2020GL089874
16. Ody, A., Thibaut, T., Berline, L., Changeux, T., André, J.-M., Chevalier, C., Blanfuné, A., Blanchot, J., Ruitton, S., Stiger-Pouvreau, V., Connan, S., Grelet, J., Aurelle, D., Guéné, M., Bataille, H., Bachelier, C., Guillemain, D., Schmidt, N., Fauvelle, V., … Ménard, F. (2019). From In Situ to satellite observations of pelagic Sargassum distribution and aggregation in the Tropical North Atlantic Ocean. PLOS ONE, 14(9), e0222584. https://doi.org/10.1371/journal.pone.0222584
17. Putman, N. F., Lumpkin, R., Olascoaga, M. J., Trinanes, J., & Goni, G. J. (2020). Improving transport predictions of pelagic Sargassum. Journal of Experimental Marine Biology and Ecology, 529. https://doi.org/10.1016/j.jembe.2020.151398
18. Robledo, D., Vázquez-Delfín, E., Freile-Pelegrín, Y., Vásquez-Elizondo, R. M., Qui-Minet, Z. N., & Salazar-Garibay, A. (2021). Challenges and Opportunities in Relation to Sargassum Events Along the Caribbean Sea. Frontiers in Marine Science, 8. https://doi.org/10.3389/fmars.2021.699664
19. Rodríguez-Martínez, R. E., Torres-Conde, E. G., & Jordán-Dahlgren, E. (2023). Pelagic Sargassum cleanup cost in Mexico. Ocean & Coastal Management, 237, 106542. https://doi.org/10.1016/j.ocecoaman.2023.106542
20. SEMAR (2024). https://digaohm.semar.gob.mx/OpSargazo/BoletinesSemanales/JULIO/Sargazo_Boletin_257_08_julio_2024.pdf
21. Uribe-Martínez, A., Berriel-Bueno, D., Chávez, V., Cuevas, E., Almeida, K. L., Fontes, J. V. H., van Tussenbroek, B. I., Mariño-Tapia, I., Liceaga-Correa, M. de los Á., Ojeda, E., Castañeda-Ramírez, D. G., & Silva, R. (2022). Multiscale distribution patterns of pelagic rafts of sargasso (Sargassum spp.) in the Mexican Caribbean (2014–2020). Frontiers in Marine Science, 9. https://doi.org/10.3389/fmars.2022.920339
22. Van Sebille, E., & Wichman, M. D. (2020). Opleiding Natuur-en Sterrenkunde Analysis of surface drifter trajectories in the tropical Atlantic to track Sargassum.
23. van Tussenbroek, B. I., Hernández Arana, H. A., Rodríguez-Martínez, R. E., Espinoza-Avalos, J., Canizales-Flores, H. M., González-Godoy, C. E., Barba-Santos, M. G., Vega-Zepeda, A., & Collado-Vides, L. (2017). Severe impacts of brown tides caused by Sargassum spp. on near-shore Caribbean seagrass communities. Marine Pollution Bulletin, 122(1–2), 272–281. https://doi.org/10.1016/j.marpolbul.2017.06.057

24. Vázquez-Delfín, E., Freile-Pelegrín, Y., Pliego-Cortés, H., & Robledo, D. (2019). Seaweed resources of Mexico: Current knowledge and future perspectives. In Botanica Marina (Vol. 62, Issue 3, pp. 275–289). De Gruyter. https://doi.org/10.1515/bot-2018-0070
25. Vázquez-Delfín E., Ávila-Velázquez V. y Robledo D. 2020. SargaZoom, Cinvestav Mérida(Version 1.1.2) [Mobile App]. Downloaded from: https://apps.apple.com/us/app/sargazoom/id1526758646
26. Wang, M., Hu, C., Barnes, B. B., Mitchum, G., Lapointe, B., & Montoya, J. P. (2019). The great Atlantic Sargassum belt. http://science.sciencemag.org/

Figure1

Click here to access/download;Figure;figure 1.tif

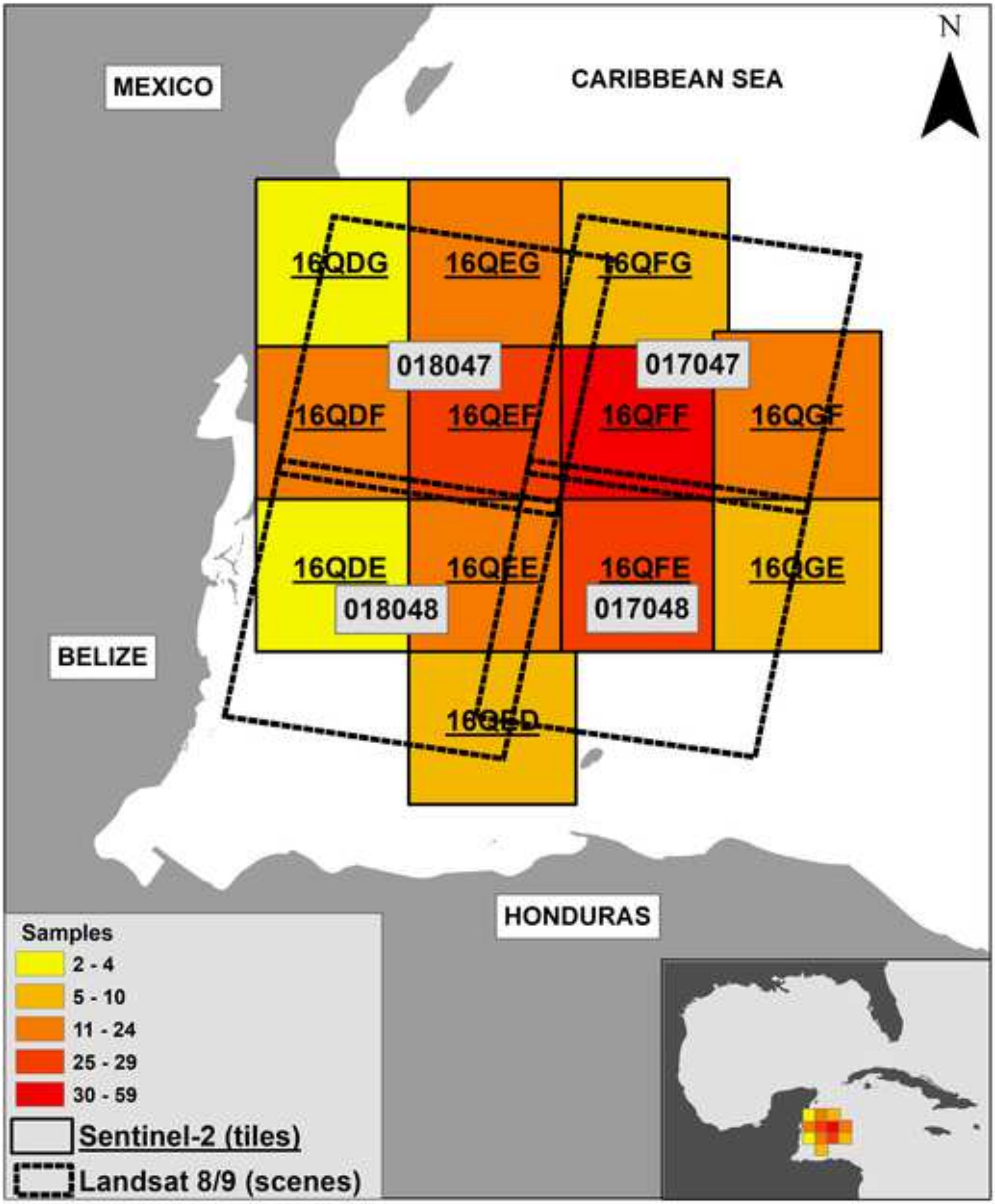

Figure2

Click here to access/download;Figure;figure 2.tif

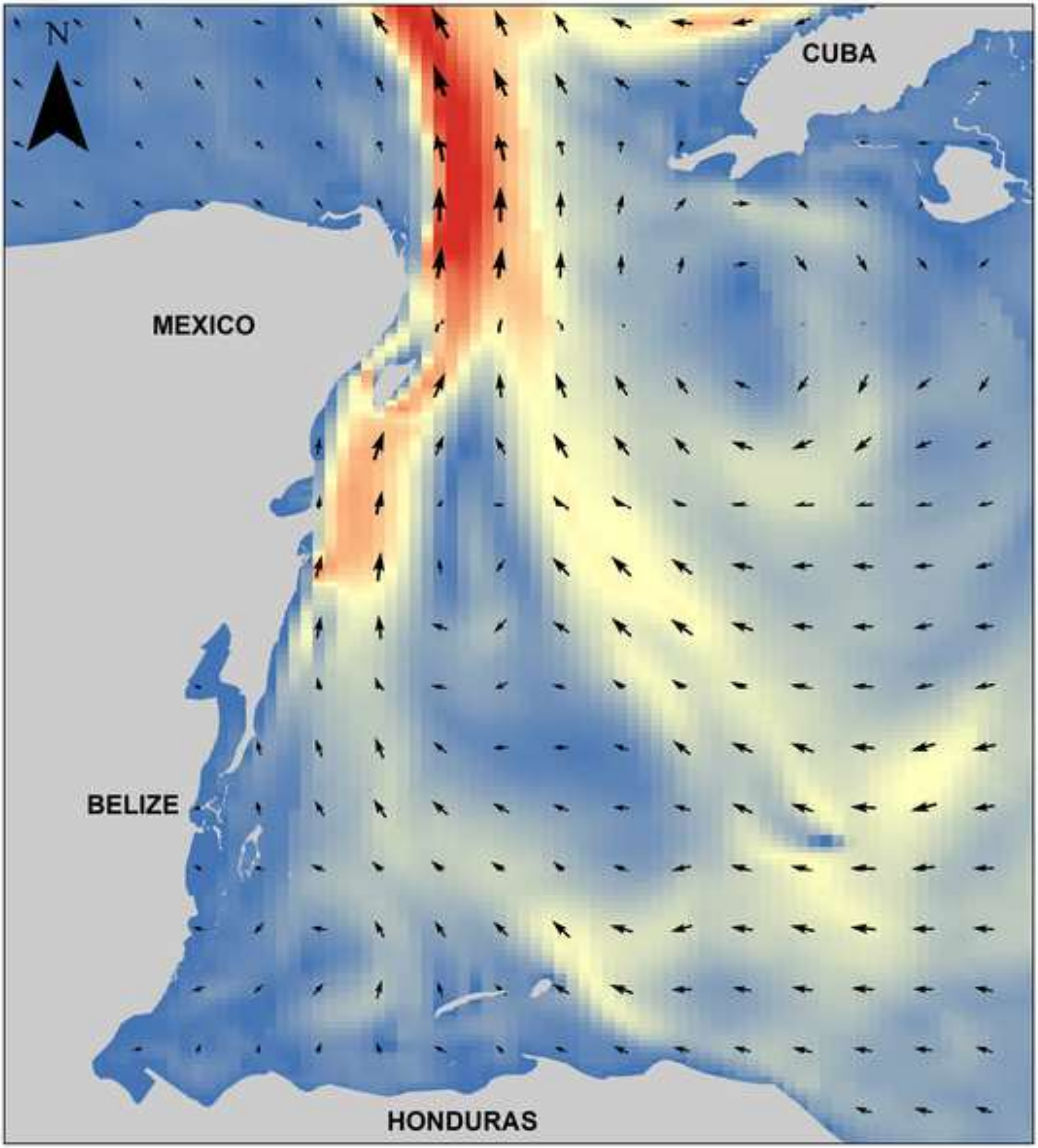

Figure3

Click here to access/download;Figure;figure 3.tif

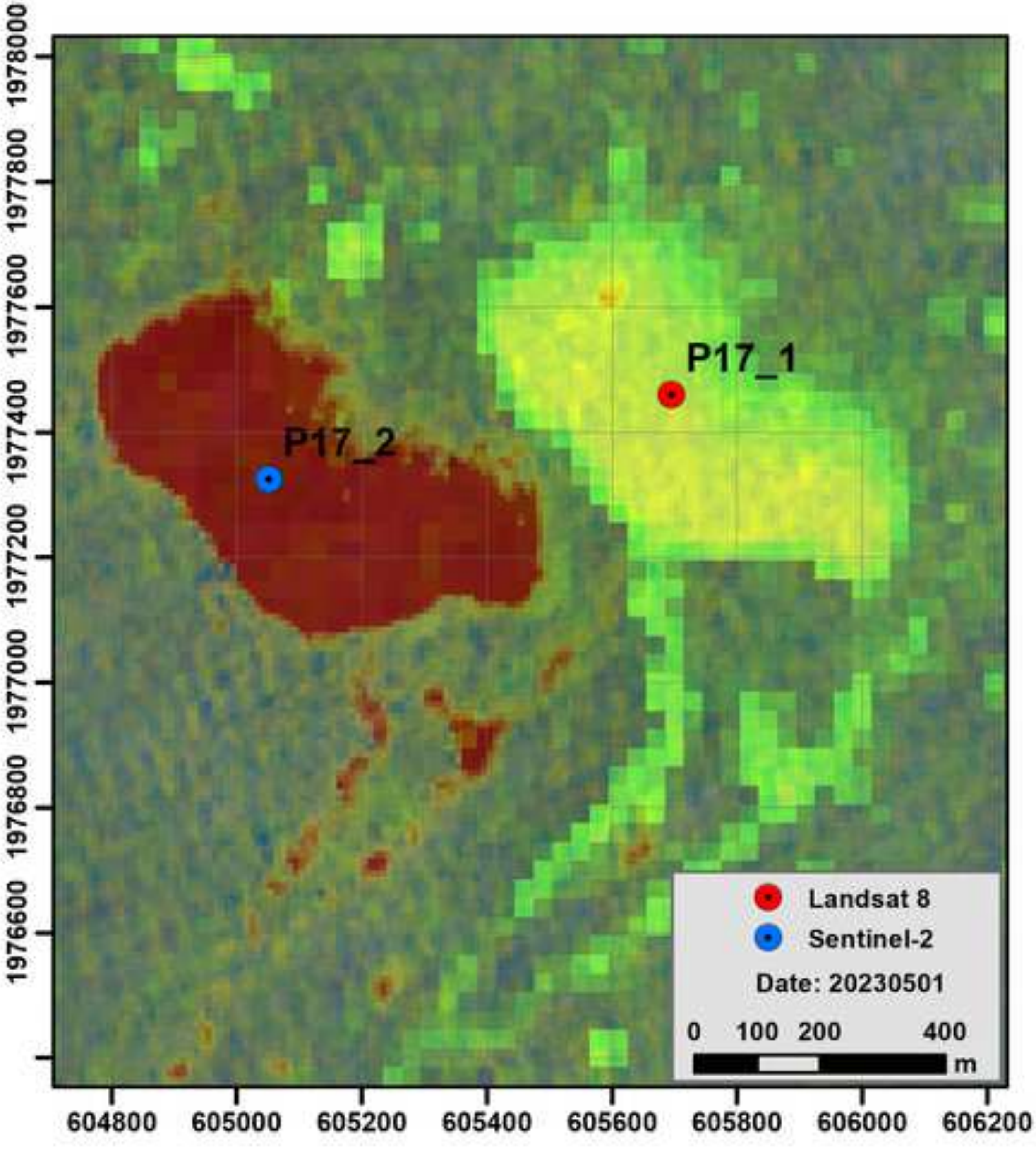

Figure4

Click here to access/download;Figure;figure 4.tif

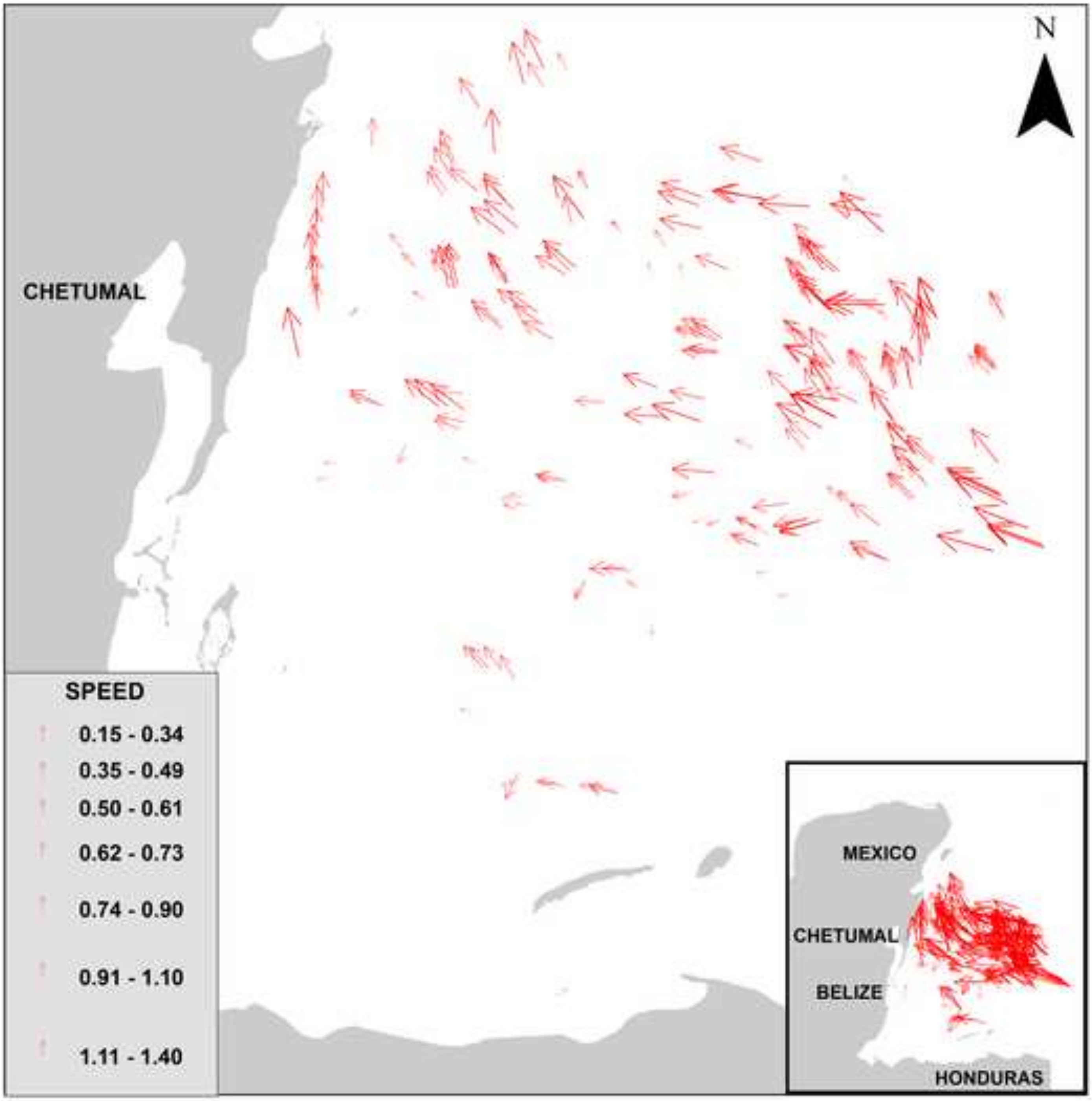

Figure5

Click here to access/download;Figure;Figure 5.tif

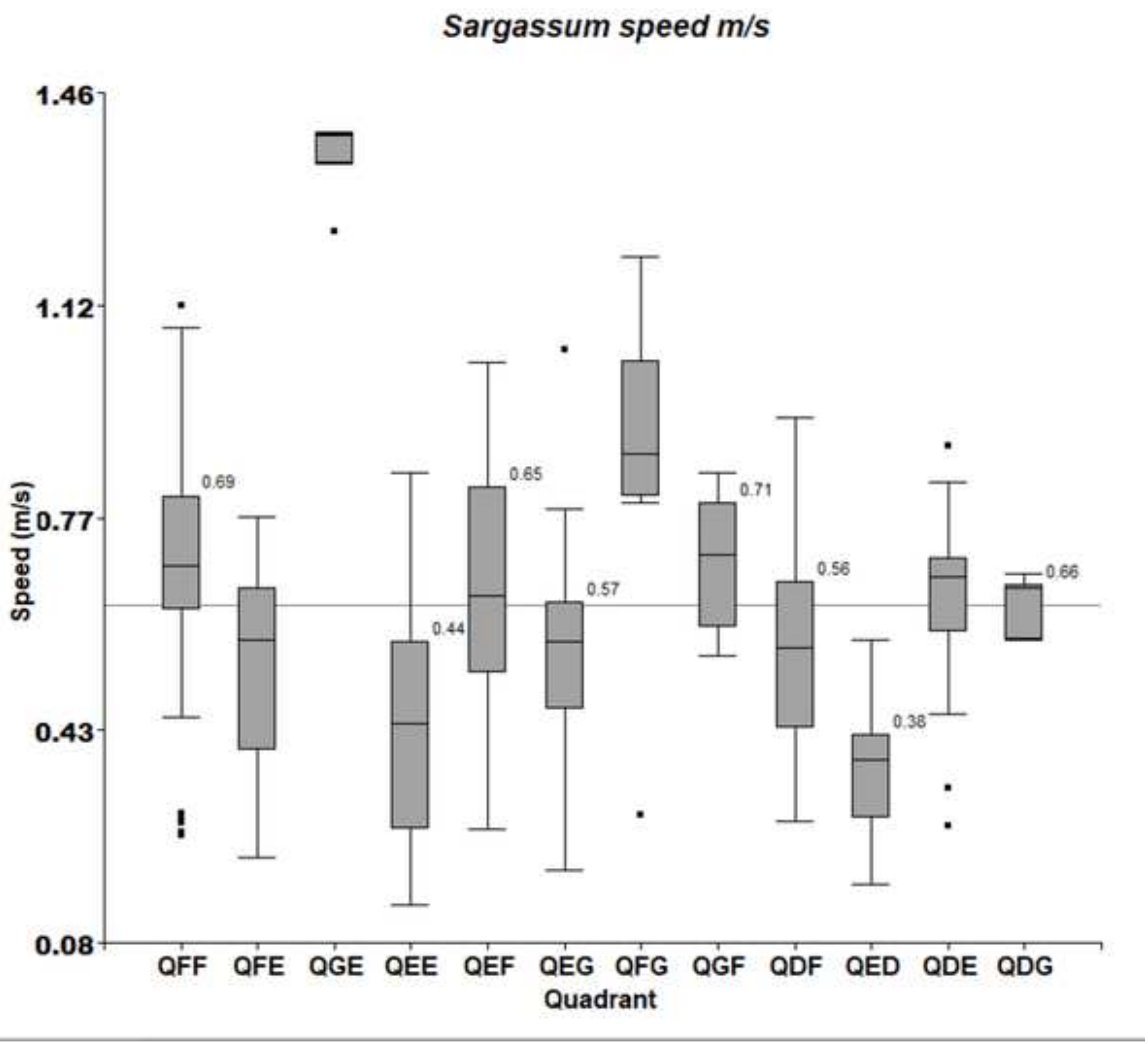

Figure6

Click here to access/download;Figure;Figure 6.tif

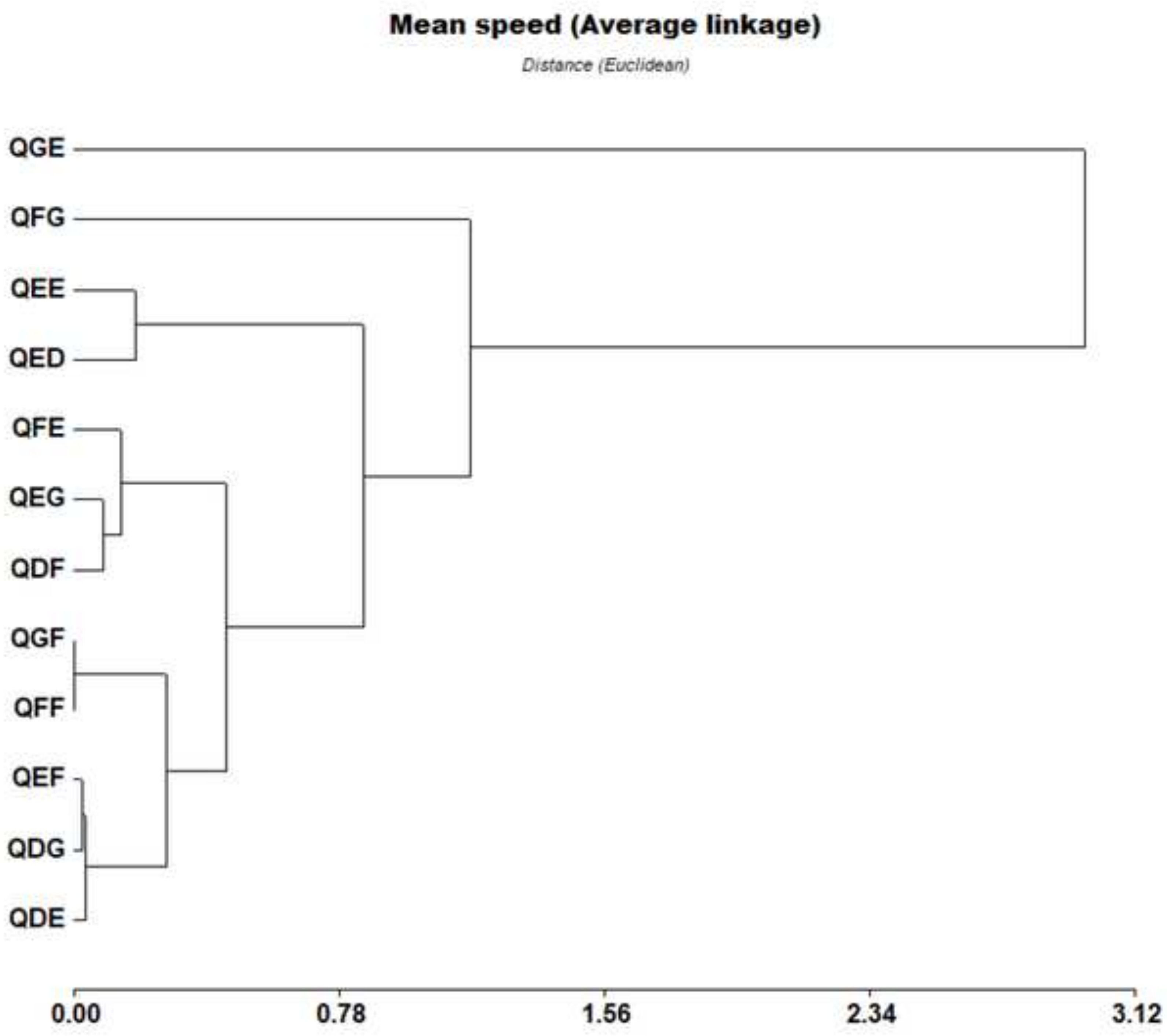

Figure7 Click here to access/download;Figure;Figure 7.tif

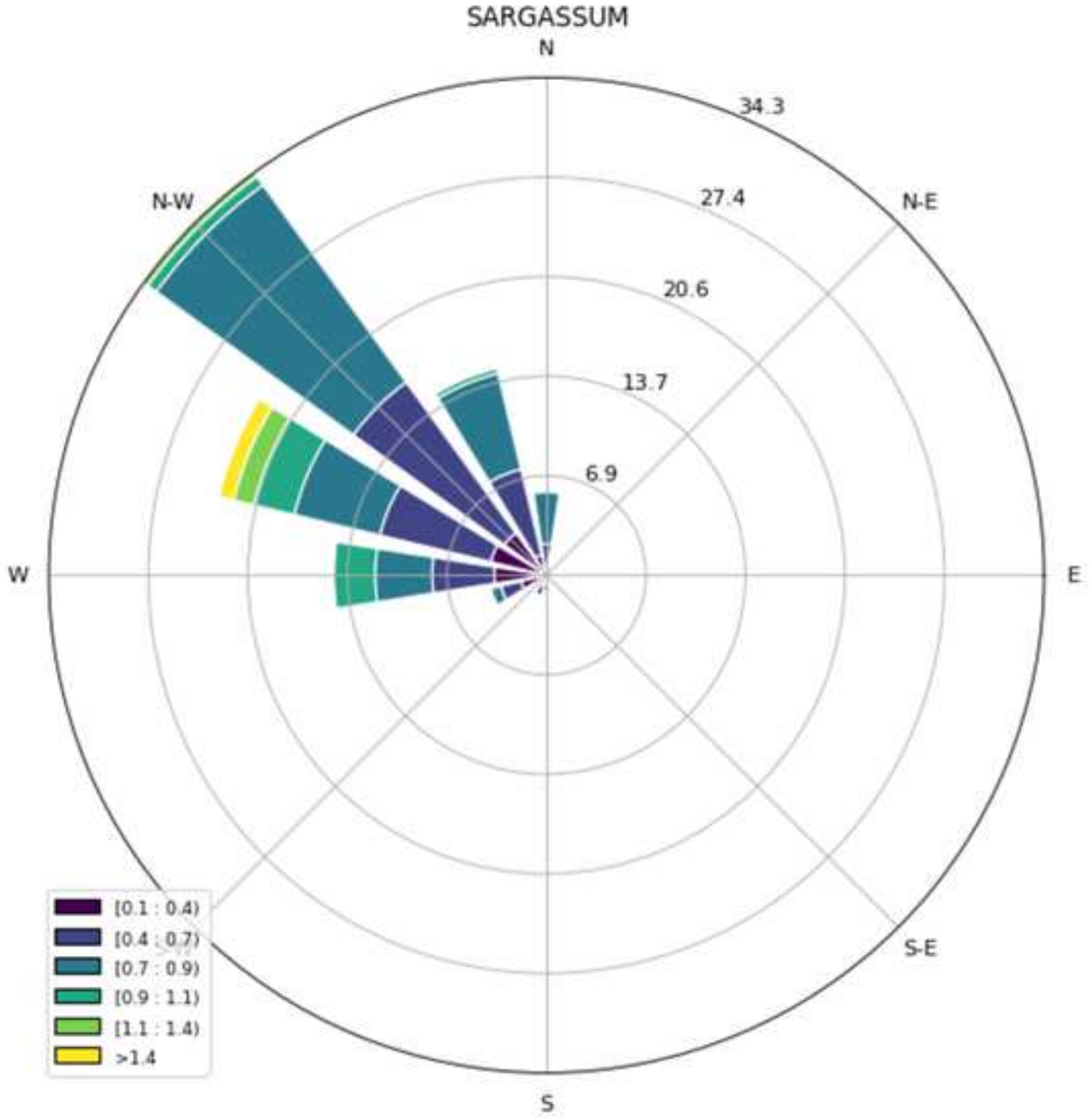

Figure8

Click here to access/download;Figure;Figure 8.tif

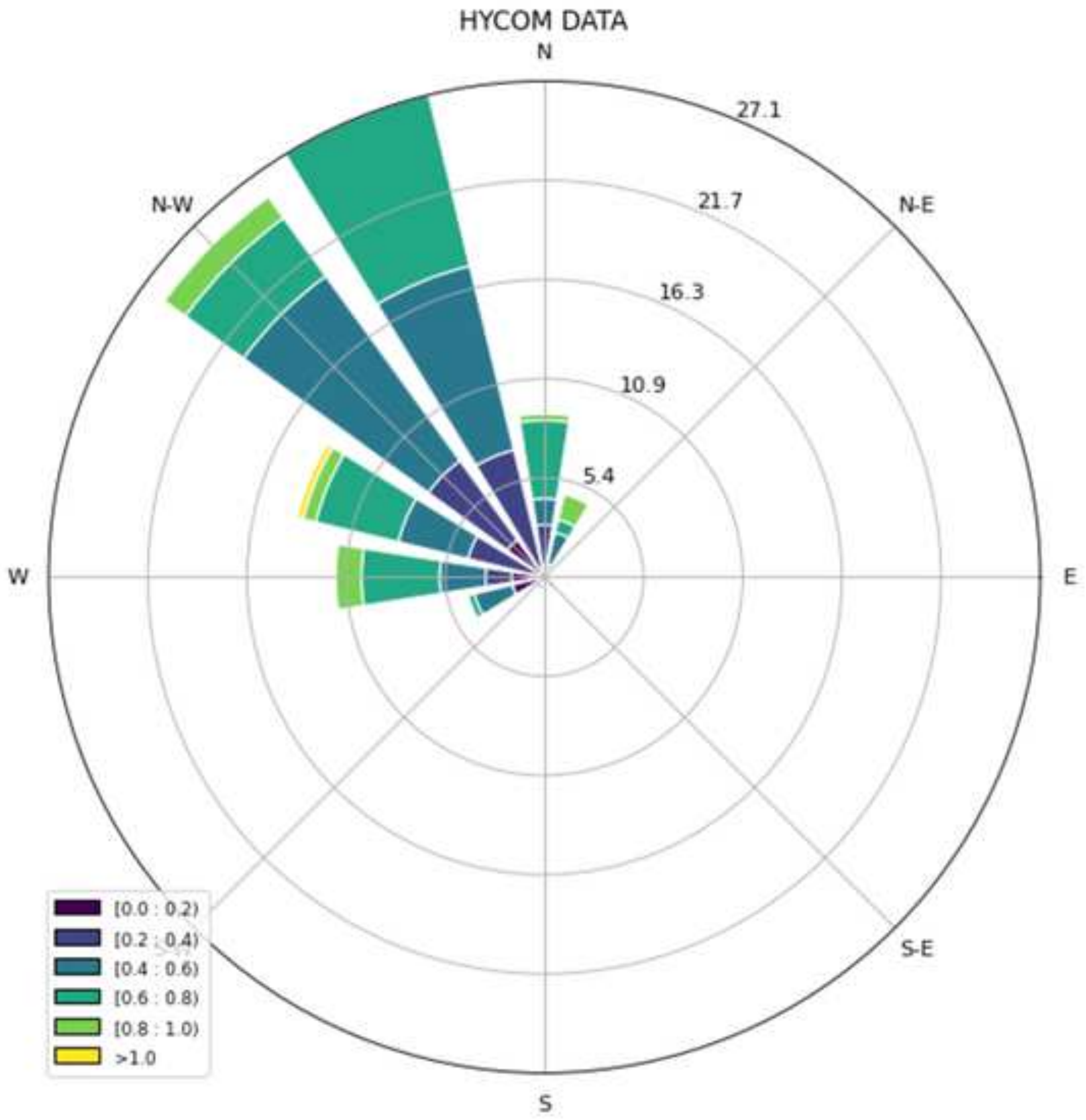

Supplement: Attachment — Submitted filename: PONE-D-24-29369_comments.pdf [file pone.0319391.s002.pdf]
