# Supplementary material for: Velocity of Sargassum migration in the Caribbean observed with Landsat 8/9 and Sentinel 2 A/B imagery
Source: PLoS One. 2025 Mar 12;20(3):e0319391. doi: 10.1371/journal.pone.0319391 (PMC11902290; doi:10.1371/journal.pone.0319391)
Supplement: Attachment — Submitted filename: Response to Reviewers.docx [file pone.0319391.s004.docx]

October 22, 2024.

Qing Wang

Academic Editor

PLOS ONE

Dear Editor,

In accordance with your instructions, this is a rebuttal letter in response to the manuscript titled:

**Velocity of Sargassum migration in the Caribbean observed with Landsat 8/9 and Sentinel 2 A/B imagery.**

**Reviewers' comments:**

Reviewer's Responses to Questions

Comments to the Author

1. Is the manuscript technically sound, and do the data support the conclusions?

The manuscript must describe a technically sound piece of scientific research with data that supports the conclusions. Experiments must have been conducted rigorously, with appropriate controls, replication, and sample sizes. The conclusions must be drawn appropriately based on the data presented.

Reviewer #1: Partly

The conclusions presented herein are based on a comprehensive analysis of LANDSAT and Sentinel 2 satellite images (L89 and S2), with the objective of identifying the velocity and direction of concentrations of Sargassum that are in motion. The method, which employs an enhanced image approach, allows for the visual selection of pixels whose geographic positions have been recorded. This enhancing approach provides high contrast among the elements of water, clouds, and Sargasso, facilitating the identification of relevant pixels for the computation of their movement.

________________________________________

2. Has the statistical analysis been performed appropriately and rigorously?

Reviewer #1: No

A probabilistic sampling design is not a viable option given the infrequent opportunities that platforms L89 and S2 can offer together to obtain close temporal (minutes) images. The high prevalence of clouds in the region, the rapid deformation of rafts over time, and the natural variability of Sargassum abundance and distribution over time represent the primary constraints. The data set for standard statistical analysis comprises 279 samples included in the S1 file and covering the period from 2019 to 2023. ________________________________________

3. Have the authors made all data underlying the findings in their manuscript fully available?

The PLOS Data policy requires authors to make all data underlying the findings described in their manuscript fully available without restriction, with rare exception (please refer to the Data Availability Statement in the manuscript PDF file). The data should be provided as part of the manuscript or its supporting information, or deposited to a public repository. For example, in addition to summary statistics, the data points behind means, medians and variance measures should be available. If there are restrictions on publicly sharing data—e.g. participant privacy or use of data from a third party—those must be specified.

Reviewer #1: Yes

Supporting information uploaded in the S1 File provides access to all data underlying the presented statistics. The Excel file contains information on the satellite platform, image name, date, time, raft latitude, raft longitude, raft velocity, raft direction, and HYCOM water velocity and direction.

_______________________________________

4. Is the manuscript presented in an intelligible fashion and written in standard English?

PLOS ONE does not copyedit accepted manuscripts, so the language in submitted articles must be clear, correct, and unambiguous. Any typographical or grammatical errors should be corrected at revision, so please note any specific errors here.

Reviewer #1: Yes

The manuscript is presented in an intelligible fashion and written in standard English with attention to language in clear academic terms to be correct and unambiguous.

________________________________________

5. Review Comments to the Author

Please use the space provided to explain your answers to the questions above. You may also include additional comments for the author, including concerns about dual publication, research ethics, or publication ethics. (Please upload your review as an attachment if it exceeds 20,000 characters)

Reviewer #1: This is quite an interesting looking at the movement of sargassum rafts across the Caribbean. However the paper lacks some level of rigour. The authors should consider the following comments to make the paper stronger:

1. Clearly this analysis goes to confirm what has already been established in literature on the role of currents in sargassum transport therefore I recommend the authors to go beyond just reporting on this link.

As stated by Miron P, Olascoaga MJ, Beron‐Vera FJ, Putman NF, Triñanes J, Lumpkin R, et al. Clustering of Marine‐Debris‐ and Sargassum ‐Like Drifters Explained by Inertial Particle Dynamics. Geophys Res Lett [Internet]. 2020 Oct 16;47(19), forecasting the paths of any floating object is inherently challenging due to the multitude of influencing factors that regulate its movement. In this regard, acquiring hard data, such as those showcased in this study, offers a solid foundation for substantiating the reality of the sargassum movement and can facilitate the evaluation and construction of predictive models to protect beaches and harvest sargassum biomass.

2. In their conclusion, the authors stated that 'Together with the dominant observed directions

in this work, scenarios of possible arrival times of Sargassum rafts at different distances and

locations can be constructed". For me with this analysis already done testing this statement could have made the work more rigorous (considering the ratfs that were detected and reported beaching-waht is the linkage?).

This proposition merits further investigation due to its intrinsic interest. Our current understanding of the project does not encompass the detection and segmentation of the total visible sargassum rafts, the prediction of future occurrences, or the collation of field data in selected marine sites and inshore locations. This is a formidable undertaking that extends beyond the scope of our current objectives.

3. The methodlogy was not presented with the rigour expected. The authors didn't go into details of how the rafts were segmented and classified beyond just mentioning a semi-automatic segmentation.

The method section was updated to include image downloading and radiometric preprocessing. RGB compositions based on reference (22), "Guillaumont B, Bajjouk T, Talec P. Seaweed and remote sensing: a critical review of sensors and data processing." In Round, FE, Chapman, DJ, editors. Progress in phycological research. Biopress Ltd; 1997. pp. 213–82. A workflow Fig 4 illustrate the main steps conducted for the rafts database (S1 File). Furthermore, the specifics of raft selection are outlined. It should be noted that a semi-automatic segmentation process was not employed in the visual analysis to select rafts and was subsequently removed from the workflow.

4. How were rafts included and excluded in the anlaysis? How were clouds handled?,etc

The presence of Sargassum rafts was determined visually using an RGB color composite based on band ratios, as proposed by (22). This composition increases the contrast between the water and the macroalgae due to algae photosynthetic activity, and clouds and shadows are well contrasted from the rafts, thus facilitating their selection.

5. There should be a clear workflow and properly explained.

A workflow figure (Fig 4) was included to provide additional support for the description of the method.

6. With some further analysis included, clear reccommendations can come out of this work.

As stated by (25), forecasting the paths of any floating object is inherently challenging due to the multitude of influencing factors that regulate its movement. In this regard, acquiring authentic data, such as those showcased in this study, offers a solid foundation for substantiating the reality of the sargassum movement and can facilitate the development and evaluation of predictive models.

7. The english language can also be improved especially with the use of tenses and cosntruction of sentences (some have been pointed out in the pdf.

Old Check sentence. Landsat 8/9 (L89) and Sentinel-2 A/B (S2) imagery were used to track velocity migration of Sargassum aggregations. Displacement characteristics provides valuable information for making preventive decisions and planning harvest the biomass.

New sentence. Imagery from Landsat 8/9 (L89) and Sentinel-2 A/B (S2) was employed to monitor the velocity migration of Sargassum aggregations. The displacement characteristics of these aggregations offer insights that can inform the formulation of preventive strategies and the planning of harvesting operations for the biomass

Old Consider re-writing this sentence.A calamity for the tourism and fishing sectors by modifying landscapes and ecosystems through the alteration of the visual quality of sandy beaches, reduction of water quality, and negative changes in the habitats of species for recreational and fishing activities.

New sentence. In addition, the alteration of landscapes and ecosystems through changes in the visual quality of sandy beaches, degradation of water quality and negative changes in the habitats of species for recreational and fishing activities has had a severe impact on the tourism and fishing sectors.

Old Consider re-writing. In their detection, satellite remote sensing through different platforms has contributed monitoring using low spatial resolution but highly synoptic platforms such as MODIS, VIIRS, and Sentinel 3 with pixel sizes of hundreds of meters and temporal resolutions of hours, and high resolution platforms such as L89 and S2 with pixels up to tens of meters and temporal resolutions of days.

New sentence. In their detection, satellite remote sensing has made a significant contribution, particularly through the use of low spatial resolution but highly synoptic platforms such as MODIS, VIIRS, and Sentinel 3, which have pixel sizes of hundreds of meters and temporal resolutions of hours. Additionally, high-resolution platforms such as L89 and S2 have been employed, with pixels up to tens of meters and temporal resolutions of days.

Old Consider re-writing What is located. It is located in the Caribbean, specifically in the Western Caribbean subregion.

New sentence. The area selected for study is situated in the Caribbean region, specifically in the Western Caribbean subregion.

Old Introduce Bounding coordinates. It is bounded by L89 tracks 17 and 18, scenes 47 and 48 according to the world reference system (WRS) of the Landsat program (https://landsat.gsfc.nasa.gov/about/the-worldwide-reference-system/ ) and those tiles corresponding to S2 (https://hls.gsfc.nasa.gov/products-description/tiling-system/ ) (Fig. 1).

New sentence. It is bounded by a rectangle defined by the following corner coordinates: upper-left (19o 43.908' N, 88o 11.070' W), upper-right (19o 28.635' N, 84o 08.2, lower-left (16o 37.619' N, 88o 11.070' W), and lower-right (16o 28.081' N, 84o 52.218' W). This area encompasses images from L89 tracks 17 and 18, scenes 47 and 48, as defined by the World Reference System (WRS) of the Landsat program (https://landsat.gsfc.nasa.gov/about/the-worldwide-reference-system/) . Additionally, it includes tiles corresponding to S2 (https://hls.gsfc.nasa.gov/products-description/tiling-system/).

Old It is not clear which platform. The platform in its submerged topography at 19°N latitude is relatively short, extending about 60km and reaching a depth of 1000 m, at the limits of the study area at a distance of 300km at a depth of 4250 m.

New sentence. The continental shelf, in its submerged topography, is relatively short, extending approximately 60 kilometers and reaching a depth of 1,000 meters. In the eastern limit of the study area, at a distance of 300 kilometers from the shoreline, the depth reaches 4,250 meters.

Old This is not clear. Images where satellites encounters occur were identified between the years 2019 to 2023.

New sentence. The search for images of the L89 and S2 satellite encounters was conducted between 2019 and 2023.

Old This need expatiation. In order to identify the rafts, RGB composite images based on quotient indices were created for both platforms, and a semi-automatic segmentation process was used by the analyst to select and match the rafts.

New text. The selection of satellite images initially focused on those acquired by L89. The image pre-processing entailed the application of the Top of Atmosphere (TOA) reflectance. The presence of Sargassum rafts was determined visually using an RGB color composite based on band ratios, as proposed by Guillaumont (1997). This composition increases the contrast between the water and the macroalgae due to algae photosynthetic activity. The search for S2 scenes from the same date or from one day before or after was then conducted. The same RGB color composite was produced on the S2 images. In a visual analysis, corresponding patches in both scenes were linked. Pairs of scenes were excluded due to the inability to identify reliable landmarks, which was attributed to the shape alterations observed in the rafts and the masking effect of cloud shadows on Sargassum patches. The linkage of rafts was recorded using the geographic position (UTM-WGS84) of landmarks on the rafts in both scenes. The preferred landmark location was taken in the central part of the patch, and in others a characteristic feature such as a corner or a discontinuity in the raft perimeter was chosen. Figure 4 illustrates the workflow used to generate the database, which is provided in the Supporting Information (S1 File).

Old It will be good to include the date in the text. The date and time of capture are relative to the center of the scene according to the metadata.

New text. The date and time (S1 File) of image capture are relative to the center of the scene according to the metadata.

Old Why not determine the center and stick to that for consistency. Otherwise how reliable are you estimates.

Response: The preferred landmark location was taken in the central part of the patch, and in others a characteristic feature such as a corner or a discontinuity in the raft perimeter. This inconsistency was attributed to the inability to identify reliable landmarks, due to the shape alterations observed in the rafts and the masking effect of cloud shadows on Sargassum patches.

Old- check sentence. This transport pattern causes tons of Sargassum biomass to reach on the coastal areas of the Mexican state of Quintana Roo, generating large accumulations in artificial net barriers placed offshore at some distance from the coastline and on the unprotected beaches.

New text. This transport pattern results in a considerable amount of Sargassum biomass reaching the coastal areas of the Mexican state of Quintana Roo, leading to the formation of extensive accumulations in artificial net barriers situated offshore and on unprotected beaches.

__________________________

6. PLOS authors have the option to publish the peer review history of their article (what does this mean?). If published, this will include your full peer review and any attached files.

If you choose “no”, your identity will remain anonymous but your review may still be made public.

Do you want your identity to be public for this peer review? For information about this choice, including consent withdrawal, please see our Privacy Policy.

Reviewer #1: No

________________________________________

[NOTE: If reviewer comments were submitted as an attachment file, they will be attached to this email and accessible via the submission site. Please log into your account, locate the manuscript record, and check for the action link "View Attachments". If this link does not appear, there are no attachment files.]

While revising your submission, please upload your figure files to the Preflight Analysis and Conversion Engine (PACE) digital diagnostic tool, https://pacev2.apexcovantage.com/. PACE helps ensure that figures meet PLOS requirements. To use PACE, you must first register as a user. Registration is free. Then, login and navigate to the UPLOAD tab, where you will find detailed instructions on how to use the tool. If you encounter any issues or have any questions when using PACE, please email PLOS at figures@plos.org. Please note that Supporting Information files do not need this step.
